# Supplementary material for: From sequence to enzyme mechanism using multi-label machine learning
Source: BMC Bioinformatics. 2014 May 19;15:150. doi: 10.1186/1471-2105-15-150 (PMC4229970; doi:10.1186/1471-2105-15-150)
Supplement: Additional file 2 — Java code of ml2db. Additional file ml2db_code.tar.gz contains the Java source code to run the multi-label machine learning experiments and save the results to database. The code’s Javadoc is included. [file 1471-2105-15-150-S2.zip › additional file 2/ml2db/ml2db/doc/main/ml2db/mulan/learn/traintest/package-summary.html]

main.ml2db.mulan.learn.traintest


JavaScript is disabled on your browser.


- Overview
- Package
- Class
- Use
- Tree
- Deprecated
- Index
- Help

- Prev Package
- Next Package

- Frames
- No Frames

- All Classes

# Package main.ml2db.mulan.learn.traintest

- Class Summary

  | Class | Description |
  | --- | --- |
  |  |  |
  | --- | --- |
  | MulanTrainExample | Give arff and xml file path, weka algorithm, cross evaluation fold and it emits learning results |
  | TrainTestEvaluator | Train/test evaluator. |
  | TrainTestEvaluatorSerialised | Class |
  | TrainTestExperimenter | Using Mulan (multi-label machine learning), this class initialises and executes Train-Test evaluation (two arff records are needed, for the training and testing files) (calling `TrainTestEvaluator`). |
  | TrainTestExperimenterSerialized | Executes a train/test evaluation using a previously trained model saved on file. |
  | TrainTestFullRun | Runs a full train/test evaluation: 1. |
  | TrainTestInputManager | An input manager that gets both the training and testing instances from a database arff record identifier. |
  | TrainTestOutputManager | Class |

- Overview
- Package
- Class
- Use
- Tree
- Deprecated
- Index
- Help

- Prev Package
- Next Package

- Frames
- No Frames

- All Classes
